# *Bmal1* deletion alters mitochondrial microstructure and function in mouse cone photoreceptors

**DOI:** 10.1016/j.isci.2025.113425

**Published:** 2025-08-22

**Authors:** Nicolas Diaz, Sondip Biswas, Wei Zhong, Khaleel Bashir, Ting Chung Suen, Jason DeBruyne, Paul Michael Iuvone, Gianluca Tosini, Hao Duong, Sharon Francis, Kenkichi Baba

**Affiliations:** 1 Department of Pharmacology and Toxicology & Neuroscience Institute, Morehouse School of Medicine, Atlanta, GA 30310, USA; 2 Department of Physiology & Cardiovascular Research Institute, Morehouse School of Medicine, Atlanta, GA 30310, USA; 3 Departments of Ophthalmology and Pharmacology & Chemical Biology, Emory University School of Medicine, Atlanta, GA 30322, USA; 4 Robert P. Apkarian Integrated Electron Microscopy Core (IEMC), Emory University School of Medicine, Atlanta, GA 30322, USA

**Keywords:** biochemistry, cell biology, specialized functions of cells

## Abstract

The mammalian retina contains an autonomous circadian system that regulates ocular physiology. The deletion of the core clock gene Bmal1 in the mouse retina disrupts retinal circuitry, alters cone spectral identity, and reduces cone viability. Cone photoreceptors have the highest energy demand among retinal neurons and are continuously exposed to high levels of oxidative stress, making them susceptible to mitochondrial dysfunction. To investigate the role of *Bmal1* in mitochondrial biology, we analyzed mitochondrial function and ultrastructure in 661W cells and mouse retinas lacking *Bmal1*. Loss of *Bmal1* impaired mitochondrial respiration, ATP production, and disrupted inner-membrane organization. Furthermore, we also identified *Mic60*, a key regulator of cristae structure as a direct transcriptional target of BMAL1. These findings highlight a critical role for Bmal1 in mitochondrial integrity and suggest a potential mechanism to explain the reduced cone viability observed in mice lacking *Bmal1*.

## Highlights

• Depletion of *Bmal1* alters mitochondrial functions and morphology in a cone-like cell line
• *Bmal1* regulates the expression of mitochondrial inner-membrane architecture protein, MIC60
• The effects of *Bmal1* deletion were also observed in the mitochondria of mouse cone cells

## Graphical abstract

**Figure:**
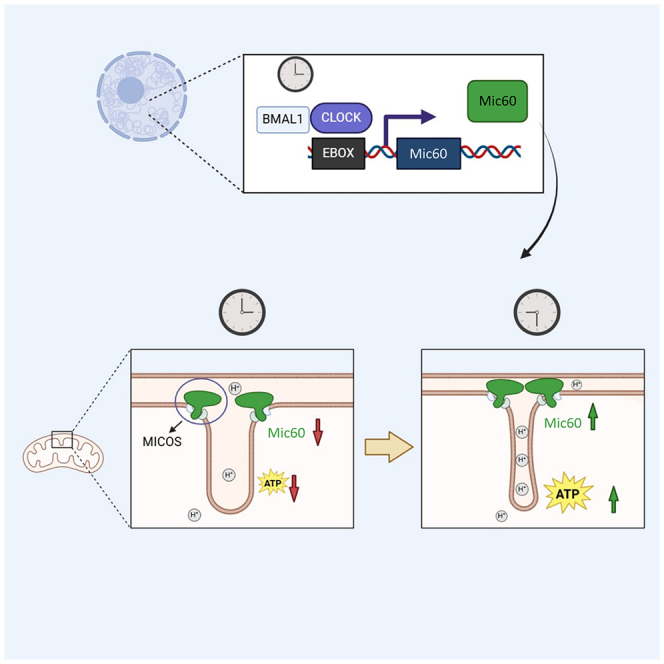

## Introduction

The retina contains its own circadian clock system that operates independently from the master clock in the brain, i.e., the suprachiasmatic nucleus of the hypothalamus.^1^^,^^2^^,^^3^^,^^4^^,^^5^ The retinal circadian clock controls a wide variety of functions within the eye, and the disruption of this circadian system has negative consequences on the functioning and viability of retinal cells.^6^ The clock gene *Bmal1* is a key component of the transcription-translation feedback loop within the molecular circadian machinery.^6^^,^^7^ Removal of *Bmal1* disrupts the molecular clockwork^8^ and induces several pathologies.^9^^,^^10^^,^^11^^,^^12^ A few studies have also investigated the effects of *Bmal1* removal on the retina, and the data indicate that its disruption affects the functioning, viability, and spectral identity of cone photoreceptors.^13^^,^^14^^,^^15^^,^^16^ Additionally, removal of *Bmal1* from the retina also affects retinal circuitry.^15^

Numerous studies have shown that *Bmal1* may regulate mitochondrial energy homeostasis and dynamics^17^^,^^18^^,^^19^ and its removal induces mitochondrial dysfunction in various cell types.^20^^,^^21^^,^^22^^,^^23^^,^^24^ Mitochondrial dysfunction is associated with reduced oxidative capacity and ATP production, increased generation of reactive oxygen species (ROS), and ROS leakage, and which may lead to various neurodegenerative and metabolic diseases.^25^^,^^26^

Cone photoreceptors are among the cells with the highest metabolic rate and energy consumption in the mammalian body.^27^^,^^28^^,^^29^ The primary energy source of cone photoreceptors is derived from large clusters of mitochondria located in the inner segments and pedicles.^30^ In the present study, we investigated the effect of *Bmal1* removal on mitochondrial functions and structure in a cone-like photoreceptor cell line (661W cells) and in a retina-specific *Bmal1* knockout mouse (rBKO, *Chx10*^*Cre*^*;Bmal1*^*fl/fl*^).^13^ Our data indicates that *Bmal1* plays an essential role in maintaining mitochondrial morphology and functions in cone cells. Furthermore, our findings suggest that the mechanisms by which *Bmal1* regulate mitochondrial morphology involve its circadian regulation of the mitochondrial inner-membrane architecture protein, MIC60.

## Results

### Removal of *Bmal1* altered mitochondrial function and glycolytic capacity of 661W cells

**Figure 1:**
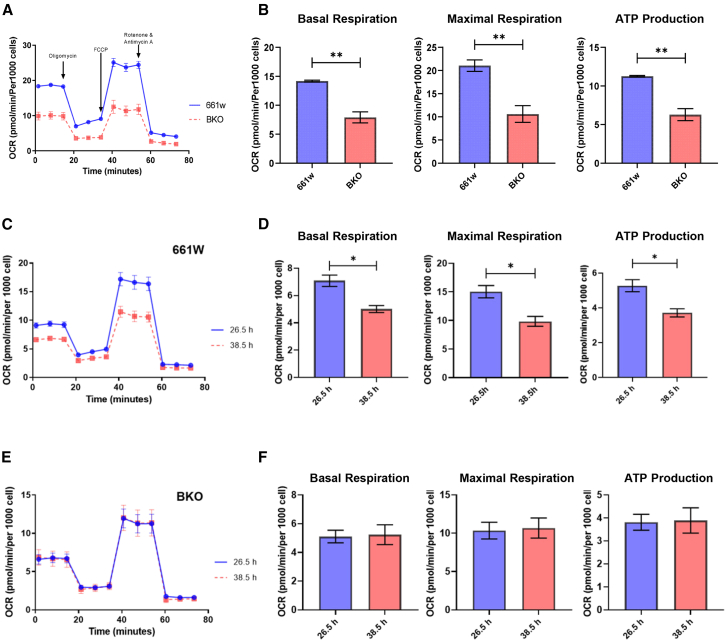
Removal of *Bmal1* altered mitochondrial function (A) Oxygen consumption rate (OCR) was measured in 661W and BKO cells using Seahorse XF HS mini analyzer with Mito stress test kit (A, mean ± SEM). (B) OCR parameters were calculated and indicated removal of *Bmal1* from 661W cells decreased basal/maximal respirations and ATP production (B, ∗∗*p* < 0.01 t test *n* = 3, mean ± SEM). (C–F) Six hundred and sixty-one cells showed circadian alteration in the level of OCR, Basal & maximal respirations, and ATP production (C and D, ∗*p* < 0.05, t test, *n* = 3) where BKO cells did not show such circadian alteration (E and F).

To evaluate the effect of *Bmal1* removal on mitochondrial functions, we first investigated oxygen consumption rate (OCR) in the cultured 661W or a *Bmal1* knockout derived from 661W cells (BKO)^31^ using Seahorse XF HS Mini analyzer with Cell Mito Stress test kit (Agilent) which measures key parameters of mitochondrial functions. The assay revealed a 2-fold decrease in the basal OCR in BKO cells (Figure 1A). The disruption of complex V activity by treating the cells with oligomycin induced a more decline in OCR in wild type 661W cells compared to BKO cells, indicating less ATP production in BKO cells. The stimulation of mitochondrial energy demand by FCCP showed that the maximum capacity of the electron transport chain was also decreased in BKO cells (Figure 1B). We then tested whether circadian variation in mitochondrial function was present in 661W and BKO cells. As shown in Figures 1C–1F, 661W cells exhibited circadian variation in basal and maximal respiration as well as ATP production, whereas no circadian variation in these parameters was observed in BKO cells (Figures 1C–1F).

### Lack of *Bmal1* affects mitochondrial morphology

**Figure 2:**
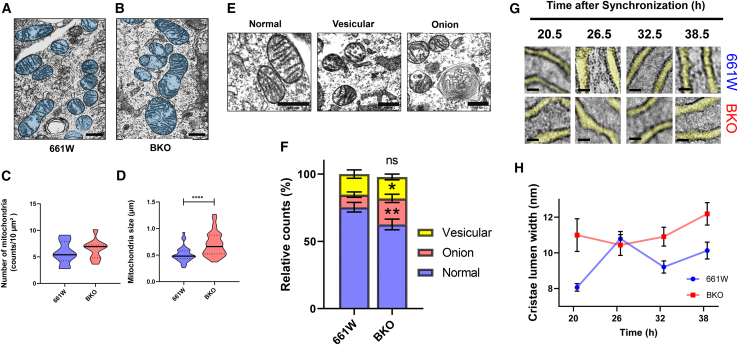
Removal of *Bmal1* affected mitochondria morphology and inner-membrane formation (A and B) The representative TEM images of mitochondria in 661W (A: scale bar 500 nm) and BKO cells (B: scale bar 500 nm). (C and D) The removal of BMAL1 did not affect the number of mitochondria in 661W cells (C); however, the mitochondrial size was slightly increased in BKO cells (D, ∗∗∗∗*p* < 0.0001, Mann–Whitney U test, *n* = 329–386). (E and F) Detailed observations using high-magnification TEM images revealed a decrease in the number of normal shaped mitochondria and an increase in onion-shaped mitochondria in BKO cells (E and F, ∗*p* < 0.05, ∗∗*p* < 0.01, Mann–Whitney U test, *n* = 25–26: scale bars 500 nm; The representative image presented in E was derived from a region within the sample shown in (A). (G) The gap width of mitochondrial cristae was measured across 24 h in 661W and BKO cells (G: scale bars 10 nm). (H) The size of the mitochondrial cristae lumen was significantly increased in BKO cells compared to 661W cells (H, *p* < 0.01 between groups, two-way ANOVA, n = 7–10 each group in each time points). Daily fluctuations in gap width were observed in 661W cells (G, *p* < 0.05, one-way ANOVA, n = 7–10), but were absent in BKO cells.

Morphological analysis of 661W and BKO cells using transmission electron microscopy (TEM) indicated that although there was no difference in the total number of mitochondria between 661W and BKO cells (Figures 2A and 2C), the size of mitochondria in the BKO cells was significantly increased with respect to that observed in 661W cells (Figures 2B and 2D). Detailed image analysis of the mitochondria morphology identified the presence of three distinct mitochondrial morphologies (e.g., normal, vesicular, and onion shape; Figure 2E) in both 661W and BKO cells. The number of onion-shaped mitochondria was significantly higher in BKO cells than in 661W cells, but no difference was observed in the number of mitochondria with vesicular shape (Figure 2F). In contrast, the number of normally shaped mitochondria was decreased in BKO cells (Figure 2F). Our morphological analysis also indicated that the cristae gap in the inner membrane of normally shaped mitochondria was significantly larger in BKO than in 661W mitochondria (Figures 2G and 2H). Additionally, 661W cells exhibited circadian fluctuations in cristae gap width, which were absent in BKO cells (Figures 2G and 2H).

### *Bmal1* regulates the circadian expression of *Mic60*

**Figure 3:**
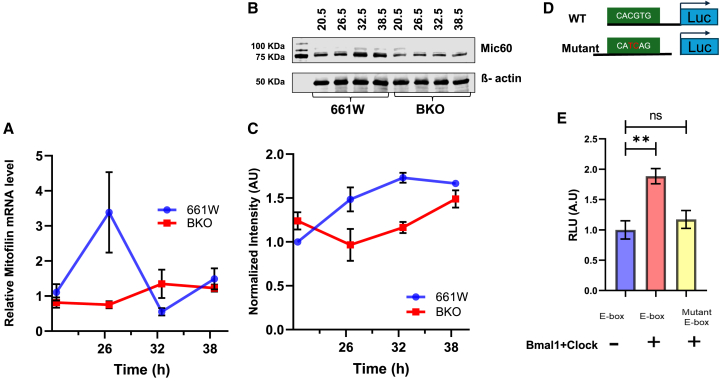
*Bmal1* regulation of circadian MICOS protein (A) The relative mRNA levels of the MICOS protein, *Mic60*, in 661W cells exhibited a robust circadian rhythm, peaking at 26.5 h post-synchronization (A, *p* < 0.05, one-way ANOVA, n = 5–6). In contrast, BKO cells did not display any rhythmicity. Additionally, the overall expression level of *Mic60* was significantly reduced in BKO cells compared to 661W cells (A), *p* < 0.05, two-way ANOVA, n = 5–6). (B and C) Western blot analysis confirmed the circadian rhythm in MIC60 protein levels in 661W cells, while no rhythmicity was observed in BKO cells (B and C, *p* < 0.0001, One-way ANOVA, *n* = 5). Moreover, MIC60 protein levels were consistently lower in BKO cells (C, *p* < 0.05, two-way ANOVA, *n* = 5). (D) To further investigate the regulatory mechanisms, a *Mic60* activation luciferase assay was performed using either an E-box or an E-box-mutated containing promoter (D, red letters indicate mutated region). (E) These assays were conducted in HEK293 cells in the presence or absence of BMAL1 and CLOCK. BMAL1 and CLOCK significantly enhanced the *Mic60* expression, whereas the mutant E-box showed no activation (E, ∗∗*p* < 0.01, One-way ANOVA followed by post hoc test, *n* = 5).

*Mic60* is a key subunit of the mitochondrial contact site and cristae organizing system (MICOS) complex, essential for maintaining cristae junctions and inner-membrane architecture.^32^^,^^33^^,^^34^ To determine whether BMAL1 influences *Mic60* levels, we investigated its expression in 661W and BKO cells. As shown in Figure 3A, *Mic60* gene expression measured by q-PCR exhibited circadian fluctuations in 661W cells, but no circadian rhythmicity was observed in BKO cells, and the overall mRNA levels were significantly lower in BKO (Figure 3A). Similarly, MIC60 protein levels showed circadian oscillations in 661W cells, which were abolished in BKO cells with a significant overall reduction in expression (Figures 3B and 3C). To further explore the contribution of BMAL1 to the regulation of *Mic60*, we analyzed its promoter region using ChIP-Atlas (Kyoto University-DBCLS). Our analysis revealed common binding sites for BMAL1, CLOCK, and REV-ERBα/β upstream and downstream of the *Mic60* transcription start site (Figure S1). To verify whether CLOCK/BMAL1 enhances *Mic60* promoter activity, we performed a luciferase promoter assay by co-expressing CLOCK and BMAL1 in 661W cells. The *Mic60* promoter (157 to +601 relative to the transcription start site) was cloned into the pGL4.13 vector containing luciferase reporter. The wild type of promoter-reporter construct containing canonical E-box was highly enhanced by the co-expression of CLOCK/BMAL1, whereas the transcriptional activity of the same promoter with a mutated E-box was not enhanced by CLOCK/BMAL1 (Figures 3D and 3E). Thus, our data indicate that the circadian clock regulates the expression of *Mic60* via CLOCK/BMAL1 action on its promoter.

### *Mic60* overexpression in *Bmal1* knockout cells rescued mitochondrial function

**Figure 4:**
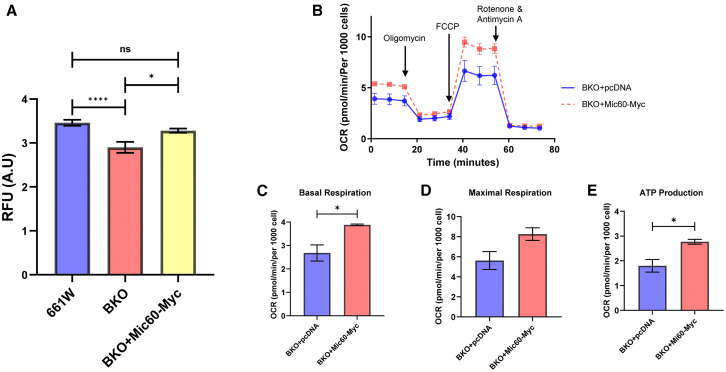
Over expression of *Mic60* rescued the membrane potential and mitochondrial function in BKO cells (A) Mitochondrial membrane potential was measured using Tetramethylrhodamine, methyl ester (TMRM). TMRM signal decreased in BKO cells, but the over expression of *Mic60* significantly increased TMRM signal (A, ∗*p* < 0.05, ∗∗∗∗*p* < 0.0001, One-way ANOVA followed by post hoc test, *n* = 6). (B–E) Mito stress test was done with *Mic60* over-expressed BKO cells (B) and *Mic60* increased the levels of basal respiration and ATP production (C–E, ∗*p* < 0.05, *t* test, *n* = 3).

Myc-tagged *Mic60*^35^ was transfected into BKO cells. Mitochondrial membrane potential was measured using tetramethylrhodamine, methyl ester (TMRM). The TMRM signal was significantly decreased in BKO cells compared to 661W cells, but the overexpression of *Mic60* in BKO cells significantly increased the TMRM signal compared to untreated BKO cells (Figure 4A), indicating that *Mic60* overexpression rescued the membrane potential in BKO cells. Mitochondrial function was also assessed in *Mic60* overexpressing BKO cells. As controls, BKO cells were transfected with a Myc-tagged pCDNA3.1 vector. Overexpression of *Mic60* increased basal respiration and ATP production levels compared to control cells (Figures 4B, 4C, and 4E). However, no statistically significant differences were observed in maximal respiration (Figure 4D) or proton leak (Figure 4B).

### Mitochondrial structure in the mouse cones

**Figure 5:**
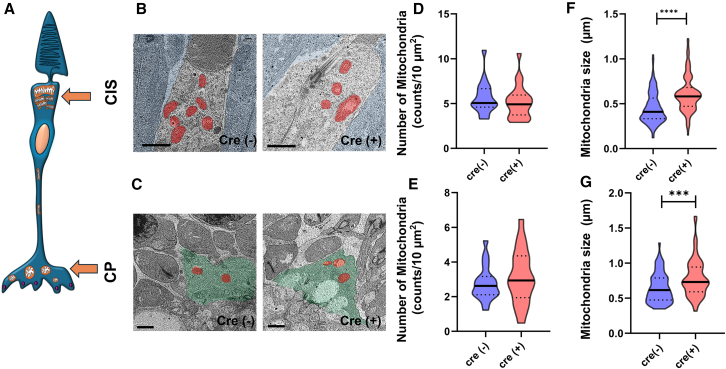
Removal of *Bmal1* enlarged mitochondria in the cone (A–C) Representative images of mitochondria (A; red) in the cone inner segment (CIS, light gray; B: scale bars 1 μm) and cone pedicle (CP, light green; C: scale bars 1 μm) were obtained from *Chx10*^*Cre*^*;Bmal1*^*fl/fl*^ (Cre+) and control (Cre−) mice. (D and E) The number of mitochondria in both the CIS and CP did not differ between the two genotypes (D and E). (F and G) However, mitochondrial size measurements revealed that mitochondria in Cre+ cones were slightly larger than those in control cones (F and G, ∗∗∗*p* < 0.001, ∗∗∗∗*p* < 0.0001, Mann–Whitney U test, *n* = 75 CP mitochondria and *n* = 155–172 mitochondria in CIS: from 3 to 4 animals).

**Figure 6:**
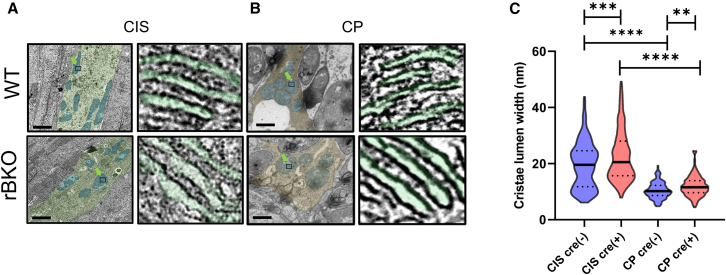
Lack of *Bmal1* in the retina increased the width of the cristae lumen in the mitochondria of mice cone (A) Representative images of mitochondrial cristae in the cone inner segments (CIS; A: scale bars 1 μm) and cone pedicles (CP; B: scale bars 1 μm) are shown. The CIS region is highlighted in light green, and the CP region is highlighted in light brown. Mitochondria are outlined in light blue, and the green arrows indicate the areas focused on for the imaging of the cristae lumen. (C) The cristae lumen width was larger in the CIS for both genotypes, and the cristae lumen of Cre+ mice was significantly larger compared to that of Cre− control mice (C, Kruskal-Wallis test, ∗∗*p* < 0.01, ∗∗∗*p* < 0.001, ∗∗∗∗*p* < 0.0001, *n* = 187–239 cristae from 3 to 4 animals).

**Figure 7:**
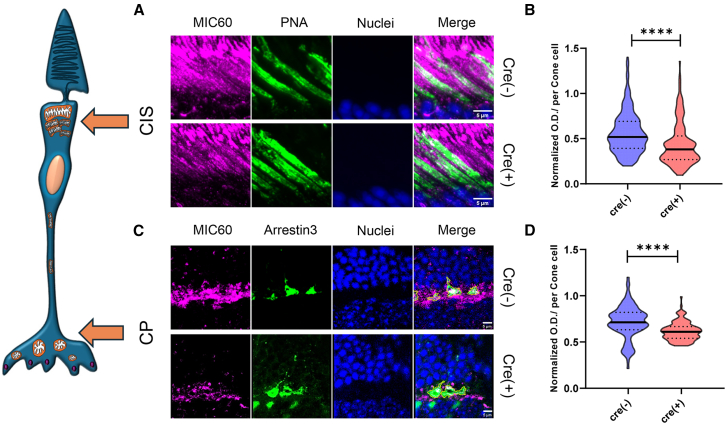
MIC60 expression was decreased in cone cells of Cre+ mice retina (A) The retina slice from Cre+ and Cre− mice was co-immunostained with PNA (Green), MIC60 (Magenta), and DAPI (blue) to visualize MIC60 expression in the cone inner-segments (CIS: A). Intensity of the MIC60 signal was quantified relative to the PNA signals. The merged area of PNA and MIC60 was pseudo colored as white (A: scale bar 5 μm). (B) Intensity of the MIC60 signal was quantified relative to the PNA signals. The level of MIC60 signal was decreased in Cre+ mice CIS (B, ∗∗∗∗*p* < 0.0001, Mann-Whitney U test, *n* = 192–282 from 6 retinas). (C) Retinal slices were co-immunostained with CONE ARRESTIN (Green), MIC60 (Magenta) and DAPI (Blue). The merged area of CONE ARRECTIN and MIC60 was pseudo colored as white (C: scale bar 5 μm). (D) Intensity of MIC60 signal was quantified relative to the CONE ARRESTIN signals. The expression level of MIC60 was approximately 20% decreased in Cre+ cones compared to control retina (D, ∗∗∗∗*p* < 0.0001, Mann-Whitney U test, *n* = 127–143 from 6 retinas).

To investigate whether the observed mitochondrial changes found in cultured cells deficient in *Bmal1* were also present in the mouse retina, mitochondrial morphology was assessed by EM in the retinal sections obtained from rBKO (*Chx10*^*Cre*^*;Bmal1*^*fl/fl*^) and control (*Bmal1*^*fl/fl*^) mice. Cone inner segments (CIS) and cone pedicles (CP), two areas known to have high mitochondria density,^36^ were investigated. No significant differences in mitochondrial number between the two genotypes in the CIS (Figures 5A, 5B, and 5D) and CP, (Figures 5A, 5C, and 5E) were observed. Consistent with the results obtained in the 661W cells (Figure 2D), the sizes of mitochondria in both areas were significantly larger in the retinas of rBKO mice than in control mice (Figures 5F and 5G). Furthermore, cristae lumen width in both the CIS and CP of rBMAL1 was increased in rBKO retinas compared to controls (Figures 6A–6C). Finally, we investigated the level of MIC60 expression in the mice cone photoreceptors obtained from rBKO and control retinas. Consistent with our observations in 661W cells (Figure 2H), the levels of MIC60 in both CP and CIS were approximately 20% lower in the rBKO retina than in control retina (Figures 7A–7D).

## Discussion

Previous work has shown that removal of *Bmal1* from the mouse retina greatly reduces the viability of cone photoreceptors during aging.^16^ Cone photoreceptors are highly energy-demanding cells that require substantial ATP expenditure^37^ and it has been reported that the circadian clock regulates mitochondrial dynamics, which are essential for maintaining optimal mitochondrial function.^17^^,^^19^^,^^38^^,^^39^ In the present study, we explored the role of *Bmal1* in the regulation of mitochondrial biology in a cone-like photoreceptor cell line (661W) and in the mouse retina. Our studies of 661W cells revealed that (i) removal of *Bmal1* negatively affects mitochondrial structure and function; (ii) the circadian clock proteins, CLOCK/BMAL1, directly controls the circadian rhythms of *Mic60* RNA; (iii) the lack of circadian rhythm in *Mic60* transcription leads to alterations of mitochondrial cristae. These effects of *Bmal1* removal in 661W cells were partially replicated *in vivo* in rBKO mouse cone photoreceptors.

Previous studies have shown that *Bmal1* plays a crucial role in mitochondrial function across various mammalian organs, influencing cellular metabolism and energy homeostasis.^17^^,^^20^^,^^23^^,^^40^ Using 661W photoreceptor-like cells, our study has shown that removal of *Bmal1* decreases in the oxygen consumption rate (OCR). Our results aligned with previous studies in hepatocytes and cardiomyocytes, which demonstrated that *Bmal1* removal increases mitochondrial size.^17^^,^^18^ This increase in mitochondrial size has been linked to elevated ROS levels.^39^ Similarly, *Bmal1* deletion in 661W cells impairs their ability to respond to oxidative stress by reducing an antioxidant capacity,^31^ consistent with these prior findings.

BKO cells have a greater number of onion-shaped mitochondria compared to 661W cells (Figure 2). Changes in mitochondrial ultrastructure can occur under various conditions,^41^^,^^42^^,^^43^ and are linked to mitochondrial function.^44^ Concentric onion-like cristae and vesicular-like structures are commonly associated with inner-membrane cristae deformation.^42^^,^^45^^,^^46^ In our study, while we observed a significant increase in onion-shaped mitochondria in BKO cells compared to 661W cells, there was no notable difference in the number of vesicular-shaped mitochondria between the two genotypes. Mitochondrial inner-membrane structure appears to be cell type-specific and undergoes regulation and remodeling in response to the cell’s energy demands.^42^^,^^43^^,^^47^ Our results suggest that *Bmal1* regulation plays a minimal role in the formation of vesicular-shaped mitochondria in this cell type.

The mitochondrial contact site and cristae organizing system (MICOS) is a protein complex crucial for the formation of mitochondrial inner-membrane cristae^41^^,^^48^ and plays a key role in regulating the electron transport chain, ATP generation, and mitochondrial DNA transcription—processes essential for mitochondrial function and cell survival.^41^^,^^46^^,^^49^ Our study suggests that cone photoreceptors increase cristae surface area to enhance ATP production, to meet the higher energy demands of cones.^36^ MIC60 (also known as IMMT), the core protein of MICOS, interacts with the outer membrane sorting and assembly machinery (SAM) to form the mitochondrial intermembrane space bridging (MIB) complex, which enriches cristae junctions.^50^^,^^51^ Numerous studies have reported that *Mic60* downregulation lead to the formation of onion-shaped mitochondria^32^^,^^42^^,^^43^^,^^52^ and an increase in mitochondrial cristae width.^53^ Thus, the increase in the gap in the cristae junctions observed in BKO cells, along with changes in *Mic60* expression, are consistent with previous experimental findings. MICOS complex is composed of six subunits which are organized into the two subcomplex into MIC10 and MIC60. It has been reported that the mutation of MIC10 has also been shown to cause dramatic alterations in cristae ultrastructure. Further investigation is needed to determine whether Bmal1 regulates MIC10 or other MICOS subunits.^54^

Our analysis of the *Mic60* promoter revealed a BMAL1/CLOCK binding site near the transcription start site (TSS). The canonical E-box motif (CACGTG) located downstream of exon 1 was assessed for its role in enhancing *Mic60* expression. We found that *Mic60* transcription was enhanced in the presence of BMAL1 and CLOCK. For many clock-regulated genes, E-box motifs are commonly located in the promoter region upstream of the TSS.^55^ However, the transcription of certain genes, including Dbp and ornithine decarboxylase (ODC), is driven by intronic E-boxes.^56^^,^^57^ Additionally, we identified another canonical E-box located +5 kb downstream of the *Mic60* TSS, before exon 2. The role of 2nd E-box is still unknown, but it may also contribute to the enhancement of *Mic60* expression.

Finally, we extended our investigation of mitochondrial structures to the cone photoreceptors of rBKO mice. Our results indicate that although we did not observe onion-like morphological changes in the rBKO retina, the size of the mitochondria is also increased in the CIS and CP of rBKO cones.

The abundance of *Mic60* appears to be highly susceptible to oxidative stress,^58^ which plays a critical role in mediating mitochondrial structure and function.^46^^,^^59^ Although 661W cells are widely used as a cultured model for cone photoreceptors, they do not fully replicate the complexity of cone photoreceptors within the functional retina of living mice. Factors such as the internal surrounding environment, including extracellular antioxidant mechanisms,^60^ and the three-dimensional structure of the retina,^61^ may contribute to structural differences in mitochondria between cultured cells and the mouse retina.

As the CIS is essential for light reception, maintaining a healthy mitochondrial structure within the CIS is crucial for cone visual function.^62^ Additionally, CIS mitochondria also function as microlenses to enhance photoreception.^63^ Cone pedicles, which provide multiple synaptic outputs to bipolar and horizontal cells, have significantly higher energy demands for neurotransmitter release compared to rod spherules.^64^ The *Bmal1* regulation of mitochondria may also contribute to these cone photo-signal transductions.^13^^,^^16^ Our findings revealed the underlying mechanism by which *Bmal1* influences mitochondrial structure and function in cone photoreceptors, illustrating the importance of the retinal clock in visual function.

### Conclusions

Our study indicates removal of *Bmal1* significantly affects mitochondrial structure and function by, at least in part, abolishing the circadian the expression level of *Mic60*. Hence, our data may support that the reduced viability of cone photoreceptors observed in the rBKO during aging^16^ may be related to the effect that *Bmal1* removal has on mitochondrial function.

### Limitations of the study

There are two technical limitations in the present study. First, as observed in many other Cre/*loxP* systems, we identified that 20–30% of Bmal1 expression remained in the retina of Chx10Cre;Bmal1fl/fl mice. Although the 70–80% depletion in Bmal1 was sufficient to produce phenotypes, the potential effects of the residual Bmal1 expression on the results remain unclear. Second, as discussed, an *in vitro* model does not necessarily represent the *in vivo* state. A cell-specific analysis of the Chx10Cre;Bmal1fl/fl mouse retina may provide further insights into the molecular mechanisms underlying the Bmal1 regulation of cone mitochondria. For instance, single-cell RNA sequencing could identify Bmal1-regulated molecules and pathways involved in the structure and function of cone mitochondria.

Our preliminary results showed no overt sex-specific differences during data collection; therefore, data from both sexes were pooled for presentation. However, if subtle sex-specific effects are observed, they will be investigated further in future studies.

## Resource availability

### Lead contact

Further information and requests for resources should be directed to and will be fulfilled by the lead contact, Kenkichi Baba (bkenkichi@msm.edu).

### Materials availability

This study did not generate new unique reagents.

### Data and code availability

• All data reported in this study will be made available through a public data repository. https://data.mendeley.com/datasets/992ssn8wvb/1.
• This article does not report original code.
• Any additional information required to reanalyze the data reported in this article is available from the lead contact upon request.

## Acknowledgments

The authors would like to thank Dr. Muayyad Al-Ubaidi (Department of Biomedical Engineering, University of Houston) for donating the 661W cells and Dr. Jean C. Bopassa (UT San Antonio Health Sciences Center) for the *Mic60*-myc plasmid. The present study was supported by 10.13039/100000002NIH, United States, grants: SC1 GM135112-01A1 to K.Baba, 1R16GM146703-01 to H.D., R21 EY031821-01A1 to G.T., GM127044 to J.P.D. and T-C.S., R01 EY004864, R21 EY035136, P30 EY006360 to P.M.I., and Research to Prevent Blindness, Challenge Grant to Emory Ophthalmology to P.M.I. The graphic abstract was created using BioRender.com.

## Author contributions

N.D., S.B., W.Z., T.-C.S., K.Bashir, H.D., and K.Baba performed experiments and analyzed experimental results. S.F., J.D., P.M.I., G.T., and K.Baba planned/designed the studies, N.D., S.B., K.Bashir, and K.Baba provided data/figures, and N.D., P.M.I., G.T., and K.Baba wrote the article.

## Declaration of interests

The authors declare no competing interests.

## STAR★Methods

### Key resources table

**Table:** 

REAGENT or RESOURCE	SOURCE	IDENTIFIER
**Antibodies**		

THE™ beta Actin Antibody, mAb, Mouse	Genscript	A00702;RRID: AB_914100
IMMT Polyclonal antibody	ProteinTech	10179-1-AP
IRDye® 800CW Goat anti-Mouse IgG Secondary Antibody	Li-Cor	926-32210;RRID: AB_621842
IRDye® 680RD Goat anti-Rabbit IgG Secondary Antibody	Li-Cor	926-68071;RRID: AB_10956166

**Bacterial and virus strains**		

Mix & Go! Competent Cells - DH5 Alpha	Zymo Research	T3007

**Chemicals, peptides, and recombinant proteins**		

TRIzol™ Reagent	Invitrogen™	15596026
DMEM, powder, high glucose	Gibco™	12100046
Isopropanol biology grade		
Ethanol 200 proof molecular biology grade		
Boston Bioproducts Inc RIPA Buffer	Boston Bioproducts Inc	BP115250ML
protease inhibitor	Cell signaling	5871
Boston Bioproducts Inc Tris Buffered Saline, TBS (20X, for Western Blot Washing)	Boston Bioproducts	BM301X1L
Goat serum	Sigma-Aldrich	G9023
Bovine Serum Albumin BSA	Sigma-Aldrich	A9418
Tetramethylrhodamine, Methyl Ester, Perchlorate (TMRM)	Invitrogen™	T668
Hoechst 33342	Thermo Scientific	62249

**Critical commercial assays**		

FlexAble CoraLite® Plus 488 Antibody Labeling Kit for Rabbit IgG	Proteintech	KFA001
FlexAble CoraLite® Plus 555 Antibody Labeling Kit for Rabbit IgG	Proteintech	KFA002
Seahorse XF Cell Mito Stress Test Kit	Agilent	103010-100
Seahorse XFp FluxPak		103022-100
iQ SYBR Green Supermix	Bio-Rad	1708880
Pierce™ BCA Protein Assay Kits	Thermo Scientific™	23225
4–20% Criterion™ TGX™ Precast Midi Protein Gel	Bio-Rad	5671095
Trans-Blot Turbo Midi 0.2 μm PVDF Transfer Packs	Bio-Rad	1704157
High-Capacity RNA-to-cDNA Kit	Applied Biosystems™	4387406
Dual-Luciferase® Reporter Assay System	Promega	E1910
Zyppy Plasmid Miniprep Kit	Zymo Research	D4019

**Experimental models: Cell lines**		

661W cells	Tan et al.	https://doi.org/10.1167/iovs.03-1114; RRID:CVCL_6240
661W Bmail1 KO cells	Baba et al.	https://doi.org/10.12688/f1000research.125133.2

**Oligonucleotides**		

Primer for IMMT/Mic60: Forward: 5′-CTGCGGGCCTGTCAGTTATC-3′ Reverse: 5′-GGAGGACGAACTTCCCACA -3′	Harvard primerbank	PrimerBank ID 26326821a1
Primers for mouse 18S: Forward: 5′-TTGTTGGTTTTCGGAACTGAGGC-3′ Reverse: 5′-GGCAAATGCTTTCGCTCTGGTC -3′	Baba et al.	https://doi.org/10.12688/f1000research.125133.2

**Recombinant DNA**		

Mouse Mic60-Myc-tagged pCDNA3.1	Tombo et al.	https://doi.org/10.1016/j.freeradbiomed.2020.06.039
pCMV10/3xflag-Clock	Addgene	#47334;RRID:Addgene_47334
pcDNA™3.1	Invitrogen™	V79020
pCAGGS-FLAG-BMAL1	Addgene	#186829;RRID:Addgene_186829
pRL-CMV Renilla Luciferase	Promega	E2261
Mic-60 WT promoter-Luciferase	This paper	
Mic-60 mutated E-box promoter-Luciferase	This paper	

**Software and algorithms**		

Integrative Genomics Viewer IGV	Broad Institute and the Regents of the University of California	https://igv.org/
ChiP-Atlas	Kyoto University with Database Center for Life Science: DBCLS; https://chip-atlas.org/	https://chip-atlas.org/
Image J	The National Institutes of Health	
Prism 8.3.0	GraphPad Software	

**Deposited data**		

Data results from the study	This paper and companion repository	https://data.mendeley.com/datasets/992ssn8wvb/1

### Experimental model and study participant details

#### Cells

661W or *Bmal1* knockout (BKO)^31^ cells were seeded in 35 mm dishes, 96-well plates (Corning), or chamber slides (Lab-Tek) at a concentration of 1 × 10^4^ to 5 × 10^5^ cells in a volume of 0.2 to 5 mL of Dulbecco’s Modified Eagle Medium (DMEM; Life Technologies) supplemented with 5% fetal bovine serum (Gibco) and 1% penicillin/streptomycin at 37°C in a 5% CO2 humidified atmosphere and grown to approximately 50% to 90% confluence, depending on the experimental protocol. The number of cells was determined using the automated cell counter (Bio-Rad TC20). The timing of consecutive cell sampling was determined based on the expression of Per2-luc rhythms obtained from cultured 661W cells, with the first peak occurring at 20.5 hours after the circadian synchronization by medium exchange.^31^ The cell lines used in this study were authenticated by western blotting. For BKO cells, the deletion of Bmal1 was regularly verified, and the results consistently confirmed their identity. Additionally, all cell lines were routinely tested and confirmed to be free of mycoplasma contamination using DAPI staining.

#### Animals

Retinal specific *Bmal1* knock-out mouse model (*Chx10*^*Cre*^*;Bmal1*^*fl/fl*^) and control (*Bmal1*^*fl/fl*^) mice on C57BL/6 background (age 3–6-month-old, male and female)^13^^,^^16^ were used in the present study. The mice were kept under standard laboratory conditions in a 12-h light/12-h dark cycle (illumination with fluorescent strip lights, 200 to 400 lx at cage level); water and food were provided *ad libitum*. Mice were genotyped by PCR analysis of genomic DNA.

Animal experimentation was carried out in accordance with the USDA and NIH Guide for the Care and Use of Laboratory Animals, and animal use protocol was approved by the Morehouse School of Medicine Veterinarian and Institutional Animal Care and Use Committee.

### Method details

#### Electron microscopy analysis

To obtain an electron microscope image of mitochondrial structure in 661W cells, cultured 661W and *Bmal1* KO cells were fixed in 2·5% glutaraldehyde in 0·1 m cacodylate buffer (pH 7·3) for 1 hour at room temperature. The cells were then rinsed with PBS for 20 minutes and postfixed with 1% aqueous OsO4 for 1 hour at room temperature. The cells were rinsed with distilled water and then en-bloc staining with 1% uranyl acetate in 0·15 M NaCl overnight at room temperature. Cells were dehydrated through graded series of ethanol and propylene oxide followed by resin infiltration and embedded with Polybed 812 resin (Polysciences). Thin sections (80 nm) were cut by RMC 7000 (Leica) equipped with a diamond knife. The sections were stained with 5% uranyl acetate followed by Reynold's lead citrate and examined with a JEOL 1200EX electron microscope. The measurements were made from both 661W and BKO cells which represent excellent cell morphology of nucleus, mitochondria, and cell membrane. For EM analysis for mouse retina, eyes were obtained from 3 months old rBKO and control mice and isolated eyeballs were fixed in 2.5% glutaraldehyde in 0.1M cacodylate buffer (pH 7.3) for 7days at room temperature. Fixed eyeballs were then stored in 0.1M sodium cacodylate buffer (pH 7.3) at 4°C until processing for TEM. On the day of preparation, retinas were dissected from eyes and cut into 300μm-thick sections with scissors. Sections were then rinsed in 0.1M sodium cacodylate buffer and post fixed in in 1% aqueous OsO4 for 1 hour at room temperature. After post fixation sections were *en bloc* stained overnight with 1% uranyl acetate at room temperature. Samples were dehydrated through graded series of ethanol and propylene oxide and then embedded in Polybed 812 resin (Electron Microscopy Sciences). One-micron thick sections were cut with a diamond knife and stained with 1% toluidine blue to choose good area for thin sectioning. Thin sections 70-80sections 70-80 nm were cut and stained with 5% uranyl acetate followed by Reynold’s lead citrate and examined in a JEOL 1400EX electron microscope at 80 kV (JEOL). For both cells and mouse retina morphological analysis, the number, size and cristae lumen width of mitochondria was evaluated from the electron micrographs with the Zeiss AxioVision LE 4.7 on PC (Zeiss). For analysis of mitochondrial cristae lumen width, the distance between the inner mitochondrial membranes of the cristae was measured using ImageJ image analysis software (NIH). For 661W cells, 7–10 images from individual culture dishes were analyzed per time point. Each image contained one or two mitochondria, and the widths of 4–12 cristae lumens per image were measured. The average cristae width per image was calculated and used for statistical analysis. Only mitochondria with normal morphology which represented approximately 60–70% of all mitochondria were selected for measurement. For mouse retina, images of mitochondria located in the inner segments and cone pedicles were obtained, and the cristae lumens visible in these regions were analyzed accordingly. All microscopic images were randomly assigned numerical identifiers by an independent lab staff, and quantitative measurements were subsequently performed in a blinded manner to eliminate observer bias.

#### Measurement of cellular oxygen consumption rate (OCR)

Cellular oxygen consumption rate of 661W and BKO cells were measured with the Seahorse Extracellular Flux XFp analyzers (Agilent). XFp Mitochondrial stress test kit (Agilent) was used to evaluate mitochondrial function according to the manufacturer’s instructions. Briefly, cells were seeded in Seahorse XF plates at a density of 12 × 10^4^ or 10 × 10^4^ cells per well and cultured for 24 h. The next day the medium was replaced with low buffered XF assay medium (103575-100 Agilent), supplemented with 10 mM glucose, unless otherwise specified, and 2 mM glutamine and cell cultures were allowed to equilibrate for 1 h at 37°C in a CO2 free incubator. The compound injection ports of the XF Assay Cartridge were loaded with oligomycin (10 mM), FCCP (7 mM) and antimycin A (40 mM) to ports A, B and C respectively. Experiments were performed at 26.5 hours after seeding. Seahorse XF analysis was performed at 37°C measuring Oxygen Consumption Rate (OCR = pmole O2/min). At the end of the analysis, the medium was removed, cells were gently washed with PBS and the number of cells in each well was measured by the cell counter (TC20, Bio-Rad).

#### Over expression of *Mic60*

Cells were seeded either in a Seahorse XFp cell culture miniplate for the Mito Stress assay or in a 96-well black-wall/clear-bottom plate for the TMRM assay. After 24 hours of seeding, cultures were transfected with the *Mic60*-Myc plasmid or the empty vector (pcDNA3.1) as a control. Plasmids were delivered using Lipofectamine 3000® (Invitrogen) according to the manufacturer's recommended protocol. The cells were cultured for an additional 24 hours, and then the transfected cells were used for the respective assay.

#### *In Silico* analysis of *Mic60* promoter

ChiP-Atlas (Kyoto University with Database Center for Life Science: DBCLS; https://chip-atlas.org/) database was used to search transcription factor binding sites within 10 kb from the *Mic60* transcription start site. PEAK Brower program was run under Integrative Genomics Viewer (IGV, Broad Institute and the Regents of the University of California).^65^^,^^66^^,^^67^ Search for binding transcription factor was done with (q<0.05) threshold and circadian related genes were searched from the results.

#### Real-time polymerase chain reaction

Cultured 661W cells were collected and the total RNA was isolated using TRIZOL (Life Technologies). cDNA was synthesized from isolated RNA using a High-Capacity RNA-to-cDNA Kit (Life Technologies). The quantitative polymerase chain reaction (q-PCR) was performed with the CFX96 Touch Real-Time PCR Detection System (Bio-Rad Laboratories, Hercules, CA, USA) using iQ SYBR Green Supermix (Bio-Rad Laboratories).

#### Western blotting analysis

Cells were grown to near confluence in 35-mm dishes or 6-well plates (Falcon), washed with ice-cold PBS, harvested in ice-cold RIPA buffer (Boston BioProducts) using a cell scraper and supplemented with protease inhibitor (Cell signaling TM #5871). Cells were transferred to 1.5 mL tube, placed on a shaker and lysed for 30 min at 4°C. Cell lysates were cleared by centrifugation (17,000 × *g* for 30 min, 4°C). The protein concentration was measured with Pierce™ BCA Protein Assay Kits (Thermo Scientific) and aliquots were mixed with Laemmli buffer, 6X SDS-Sample buffer, (BP-11R, Boston Bioproducts), heated to 95°C, 10 μg of protein was loaded per well on a Criterion Midi Protein Gel with the Criterion Vertical Electrophoresis Cell System (Bio-Rad Laboratories). Proteins were transferred by using the Trans-Blot Turbo Mini 0.2 μm PVDF Transfer Packs (Bio-Rad Laboratories.) with the Bio-Rad Trans-Blot Turbo Transfer system. Membranes were washed with 1X Tris buffered saline (TBS), diluted from a 20X TBS stock solution (Boston Bioproducts) for 5 minutes. Non-specific binding was blocked by 5% non-fat dry milk in TBS. The primary antibody in TBST (0.1% Tween-20 and 2.5% non-fat dry milk in TBS) was then added to the membrane and placed on a rocking platform at 4°C overnight. The membrane was washed in TBST three times for 10 minutes on a rotating platform at room temperature. Then the secondary antibody (1:20000 IRDye® 680RD Goat anti-Rabbit IgG Secondary Antibody and IRDye® 800 CW Goat anti-Mouse IgG Secondary Antibody, LI-COR) in TBST was added. After 1-hour incubation at room temperature, the membrane was washed in TBST three times for 10 minutes each time, after which imaging was done with the LI-COR Odyssey infrared scanner Imaging system (LI-COR).

#### Immunohistochemistry

Mouse eyes were fixed with paraformaldehyde (PFA) 4% and cryoprotected with 30 % sucrose. 14 μm cryosections containing retina were made from mouse eye using cryostat (Epredia™ HM525 NX). After 5% Normal Goat Serum, 1% BSA and 0.4% Triton-X100 in PBS was used to permeabilize tissues and block unspecific binding, sections were incubated overnight at 4°C with a mix of IMMT (MIC60: Proteintech 10179-1-AP 1:1000) and ARRESTIN 3 (Millipore AB15282 1:1000 ) antibodies. Primaries antibodies were labeled using Proteintech FlexAble Antibody labeling Kit (KFA001, KFA002) prior to incubate samples. Cones inner segments were stained using Peanut Agglutinin (PNA), Rhodamine (RL-1072, Vector Labs 1:1000) After washing with PBS, slides were mounted with ProLong™ Gold Antifade Mountant with DAPI (Invitrogen™) and retinal sections were visualized with a fluorescence microscope (Zeiss LSM700).

#### Promoter assay

HEK293Cells were seeded in 24 well dishes, 24 hours later, were transfected with 10 ng of *Mic60* promoter-GL4.13 or Mutated *Mic60* promoter-GL4.13, 1 ng of pRL-CMV Vector (Promega E226A), along with 150 ng of Bmal1-CMV-pcDNA3.1, CLOCK-CMV-pcDNA3.1. Appropriate quantity of pcDNA3.1 empty vector was added to have a total of 500 ng of DNA. Lipofectamine 3000® (Invitrogen) was used to deliver plasmid into the cells. 24 hours after transfection luminescence was measured using Dual-Luciferase® Reporter Assay System (Promega E1910) in a BioTek Cytation 3 plate reader.

#### TMRM fluorescence measurement

Cells were seeded into Corning® 96-well Flat Clear Bottom Black (corning 3904) and transfected previously described. After 24 hours of transfection cells were incubated with 0.5 μM Tetramethylrhodamine, Methyl Ester, Perchlorate (TMRM) (Invitrogen, T668) for 30 minutes 37°C, in 5% CO_2_ incubator. Cells were rinsed and changed to non- Phenol Red DMEM. Fluorescence was measured in Cytation 3 plate reader (EX:545/EM 590). To normalize reading cultures were fixed using formalin 4% in PBS for 10 minutes, room temperature. After fixation cells were stained with Hoechst 33342 (Thermo Scientific 62249) and fluorescence was measured (EX:350/EM:460).

### Quantification and statistical analysis

All bar and line graph data are presented as mean ± SEM, with sample sizes determined based on prior studies from our laboratories and established standards in cellular and molecular neuroscience. In violin plots, the solid line indicates the median, and the dotted lines represent the interquartile range. Collected data were evaluated for normality with Shapiro-Wilk test and data set were analyzed with either Mann–Whitney U test or unpaired t–test depending on the data distribution. One-way and Two-way ANOVA followed by the Tukey multiple comparison test was also used to evaluate multiple groups. Statistically significant differences identified through the analyses are indicated in the figures as follows: ∗p < 0.05, ∗∗p < 0.01, ∗∗∗p < 0.001, and ∗∗∗∗p < 0.0001. Comparisons that did not reach statistical significance are labeled as “n.s.” (not significant). Detailed descriptions of the statistical tests used for each comparison are provided in the corresponding figure legends. All statistics were performed, and graphs were made with the computer software, Prism 8.3.0 (GraphPad).

## References

[1] Besharse J.C., Iuvone P.M. (1983). Circadian clock in Xenopus eye controlling retinal serotonin N-acetyltransferase. Nature.

[2] Tosini G., Menaker M. (1996). Circadian rhythms in cultured mammalian retina. Science.

[3] Tosini G., Pozdeyev N., Sakamoto K., Iuvone P.M. (2008). The circadian clock system in the mammalian retina. Bioessays.

[4] McMahon D.G., Iuvone P.M., Tosini G. (2014). Circadian organization of the mammalian retina: from gene regulation to physiology and diseases. Prog. Retin. Eye Res..

[5] DeVera C., Baba K., Tosini G. (2019). Retinal circadian clocks are major players in the modulation of retinal functions and photoreceptor viability. Yale J. Biol. Med..

[6] Hogenesch J.B., Chan W.K., Jackiw V.H., Brown R.C., Gu Y.Z., Pray-Grant M., Perdew G.H., Bradfield C.A. (1997). Characterization of a subset of the basic-helix-loop-helix-PAS superfamily that interacts with components of the dioxin signaling pathway. J. Biol. Chem..

[7] Ikeda M., Nomura M. (1997). cDNA cloning and tissue-specific expression of a novel basic helix-loop-helix/PAS protein (BMAL1) and identification of alternatively spliced variants with alternative translation initiation site usage. Biochem. Biophys. Res. Commun..

[8] Bunger M.K., Wilsbacher L.D., Moran S.M., Clendenin C., Radcliffe L.A., Hogenesch J.B., Simon M.C., Takahashi J.S., Bradfield C.A. (2000). Mop3 is an essential component of the master circadian pacemaker in mammals. Cell.

[9] Kondratov R.V., Kondratova A.A., Gorbacheva V.Y., Vykhovanets O.V., Antoch M.P. (2006). Early aging and age-related pathologies in mice deficient in BMAL1, the core component of the circadian clock. Genes Dev..

[10] Maury E., Ramsey K.M., Bass J. (2010). Circadian rhythms and metabolic syndrome: from experimental genetics to human disease. Circ. Res..

[11] Evans J.A., Davidson A.J. (2013). Health consequences of circadian disruption in humans and animal models. Prog. Mol. Biol. Transl. Sci..

[12] Musiek E.S., Lim M.M., Yang G., Bauer A.Q., Qi L., Lee Y., Roh J.H., Ortiz-Gonzalez X., Dearborn J.T., Culver J.P. (2013). Circadian clock proteins regulate neuronal redox homeostasis and neurodegeneration. J. Clin. Investig..

[13] Storch K.F., Paz C., Signorovitch J., Raviola E., Pawlyk B., Li T., Weitz C.J. (2007). Intrinsic circadian clock of the mammalian retina: importance for retinal processing of visual information. Cell.

[14] Sawant O.B., Horton A.M., Zucaro O.F., Chan R., Bonilha V.L., Samuels I.S., Rao S. (2017). The circadian clock gene Bmal1 controls thyroid hormone-mediated spectral identity and cone photoreceptor function. Cell Rep..

[15] Baba K., Ribelayga C.P., Michael Iuvone P., Tosini G. (2018). The retinal circadian clock and photoreceptor viability. Adv. Exp. Med. Biol..

[16] Baba K., Piano I., Lyuboslavsky P., Chrenek M.A., Sellers J.T., Zhang S., Gargini C., He L., Tosini G., Iuvone P.M. (2018). Removal of clock gene Bmal1 from the retina affects retinal development and accelerates cone photoreceptor degeneration during aging. Proc. Natl. Acad. Sci. USA.

[17] Jacobi D., Liu S., Burkewitz K., Kory N., Knudsen N.H., Alexander R.K., Unluturk U., Li X., Kong X., Hyde A.L. (2015). Hepatic Bmal1 regulates rhythmic mitochondrial dynamics and promotes metabolic fitness. Cell Metab..

[18] Li E., Li X., Huang J., Xu C., Liang Q., Ren K., Bai A., Lu C., Qian R., Sun N. (2020). BMAL1 regulates mitochondrial fission and mitophagy through mitochondrial protein BNIP3 and is critical in the development of dilated cardiomyopathy. Protein Cell.

[19] Ye P., Li W., Huang X., Zhao S., Chen W., Xia Y., Yu W., Rao T., Ning J., Zhou X. (2022). BMAL1 regulates mitochondrial homeostasis in renal ischaemia-reperfusion injury by mediating the SIRT1/PGC-1α axis. J. Cell Mol. Med..

[20] Peek C.B., Affinati A.H., Ramsey K.M., Kuo H.Y., Yu W., Sena L.A., Ilkayeva O., Marcheva B., Kobayashi Y., Omura C. (2013). Circadian clock NAD+ cycle drives mitochondrial oxidative metabolism in mice. Science.

[21] Oliva-Ramírez J., Moreno-Altamirano M.M.B., Pineda-Olvera B., Cauich-Sánchez P., Sánchez-García F.J. (2014). Crosstalk between circadian rhythmicity, mitochondrial dynamics and macrophage bactericidal activity. Immunology.

[22] Nohara K., Mallampalli V., Nemkov T., Wirianto M., Yang J., Ye Y., Sun Y., Han L., Esser K.A., Mileykovskaya E. (2019). Nobiletin fortifies mitochondrial respiration in skeletal muscle to promote healthy aging against metabolic challenge. Nat. Commun..

[23] Alexander R.K., Liou Y.H., Knudsen N.H., Starost K.A., Xu C., Hyde A.L., Liu S., Jacobi D., Liao N.S., Lee C.H. (2020). Bmal1 integrates mitochondrial metabolism and macrophage activation. eLife.

[24] Quattrocelli M., Wintzinger M., Miz K., Levine D.C., Peek C.B., Bass J., McNally E.M. (2022). Muscle mitochondrial remodeling by intermittent glucocorticoid drugs requires an intact circadian clock and muscle PGC1α. Sci. Adv..

[25] Guo C., Sun L., Chen X., Zhang D. (2013). Oxidative stress, mitochondrial damage and neurodegenerative diseases. Neural Regen. Res..

[26] Aguilar-López B.A., Moreno-Altamirano M.M.B., Dockrell H.M., Duchen M.R., Sánchez-García F.J. (2020). Mitochondria: an integrative hub coordinating circadian rhythms, metabolism, the microbiome, and immunity. Front. Cell Dev. Biol..

[27] Anderson B., Saltzman H.A. (1964). Retinal oxygen utilization measured by hyperbaric blackout. Arch. Ophthalmol..

[28] Yu D.Y., Cringle S.J. (2001). Oxygen distribution and consumption within the retina in vascularised and avascular retinas and in animal models of retinal disease. Prog. Retin. Eye Res..

[29] Ingram N.T., Fain G.L., Sampath A.P. (2020). Elevated energy requirement of cone photoreceptors. Proc. Natl. Acad. Sci. USA.

[30] Hayes M.J., Tracey-White D., Kam J.H., Powner M.B., Jeffery G. (2021). The 3D organisation of mitochondria in primate photoreceptors. Sci. Rep..

[31] Baba K., Suen T.C., Goyal V., Stowie A., Davidson A., DeBruyne J., Tosini G. (2022). The circadian clock mediates the response to oxidative stress in a cone photoreceptor-like (661W) cell line via regulation of glutathione peroxidase activity. F1000Res..

[32] John G.B., Shang Y., Li L., Renken C., Mannella C.A., Selker J.M.L., Rangell L., Bennett M.J., Zha J. (2005). The mitochondrial inner membrane protein mitofilin controls cristae morphology. Mol. Biol. Cell.

[33] Zerbes R.M., van der Klei I.J., Veenhuis M., Pfanner N., van der Laan M., Bohnert M. (2012). Mitofilin complexes: conserved organizers of mitochondrial membrane architecture. Biol. Chem..

[34] von der Malsburg K., Müller J.M., Bohnert M., Oeljeklaus S., Kwiatkowska P., Becker T., Loniewska-Lwowska A., Wiese S., Rao S., Milenkovic D. (2011). Dual role of mitofilin in mitochondrial membrane organization and protein biogenesis. Dev. Cell.

[35] Tombo N., Imam Aliagan A.D., Feng Y., Singh H., Bopassa J.C. (2020). Cardiac ischemia/reperfusion stress reduces inner mitochondrial membrane protein (Mitofilin) levels during early reperfusion. Free Radic. Biol. Med..

[36] Perkins G.A., Ellisman M.H., Fox D.A. (2003). Three-dimensional analysis of mouse rod and cone mitochondrial cristae architecture: bioenergetic and functional implications. Mol. Vis..

[37] Kawamura S., Tachibanaki S. (2008). Rod and cone photoreceptors: molecular basis of the difference in their physiology. Comp. Biochem. Physiol. Mol. Integr. Physiol..

[38] Schmitt K., Grimm A., Dallmann R., Oettinghaus B., Restelli L.M., Witzig M., Ishihara N., Mihara K., Ripperger J.A., Albrecht U. (2018). Circadian control of DRP1 activity regulates mitochondrial dynamics and Bioenergetics. Cell Metab..

[39] Peng T.I., Jou M.J. (2004). Mitochondrial swelling and generation of reactive oxygen species induced by photoirradiation are heterogeneously distributed. Ann. N. Y. Acad. Sci..

[40] Peek C.B., Levine D.C., Cedernaes J., Taguchi A., Kobayashi Y., Tsai S.J., Bonar N.A., McNulty M.R., Ramsey K.M., Bass J. (2017). Circadian clock interaction with HIF1α mediates oxygenic metabolism and anaerobic glycolysis in skeletal muscle. Cell Metab..

[41] Mukherjee I., Ghosh M., Meinecke M. (2021). MICOS and the mitochondrial inner membrane morphology - when things get out of shape. FEBS Lett..

[42] Vincent A.E., Ng Y.S., White K., Davey T., Mannella C., Falkous G., Feeney C., Schaefer A.M., McFarland R., Gorman G.S. (2016). The spectrum of mitochondrial ultrastructural defects in mitochondrial myopathy. Sci. Rep..

[43] Klecker T., Westermann B. (2021). Pathways shaping the mitochondrial inner membrane. Open Biol..

[44] Jiang D., Gao F., Zhang Y., Wong D.S.H., Li Q., Tse H.F., Xu G., Yu Z., Lian Q. (2016). Mitochondrial transfer of mesenchymal stem cells effectively protects corneal epithelial cells from mitochondrial damage. Cell Death Dis..

[45] Pánek T., Eliáš M., Vancová M., Lukeš J., Hashimi H. (2020). Returning to the fold for lessons in mitochondrial crista diversity and evolution. Curr. Biol..

[46] Hu C., Shu L., Huang X., Yu J., Li L., Gong L., Yang M., Wu Z., Gao Z., Zhao Y. (2020). OPA1 and MICOS regulate mitochondrial crista dynamics and formation. Cell Death Dis..

[47] Zick M., Rabl R., Reichert A.S. (2009). Cristae formation-linking ultrastructure and function of mitochondria. Biochim. Biophys. Acta.

[48] Li H., Ruan Y., Zhang K., Jian F., Hu C., Miao L., Gong L., Sun L., Zhang X., Chen S. (2016). Mic60/Mitofilin determines MICOS assembly essential for mitochondrial dynamics and mtDNA nucleoid organization. Cell Death Differ..

[49] Friedman J.R., Mourier A., Yamada J., McCaffery J.M., Nunnari J. (2015). MICOS coordinates with respiratory complexes and lipids to establish mitochondrial inner membrane architecture. eLife.

[50] Ott C., Ross K., Straub S., Thiede B., Götz M., Goosmann C., Krischke M., Mueller M.J., Krohne G., Rudel T., Kozjak-Pavlovic V. (2012). Sam50 functions in mitochondrial intermembrane space bridging and biogenesis of respiratory complexes. Mol. Cell Biol..

[51] Stephan T., Brüser C., Deckers M., Steyer A.M., Balzarotti F., Barbot M., Behr T.S., Heim G., Hübner W., Ilgen P. (2020). MICOS assembly controls mitochondrial inner membrane remodeling and crista junction redistribution to mediate cristae formation. EMBO J..

[52] Tsai P.I., Lin C.H., Hsieh C.H., Papakyrikos A.M., Kim M.J., Napolioni V., Schoor C., Couthouis J., Wu R.M., Wszolek Z.K. (2018). PINK1 phosphorylates MIC60/mitofilin to control structural plasticity of mitochondrial crista junctions. Mol. Cell.

[53] Glytsou C., Calvo E., Cogliati S., Mehrotra A., Anastasia I., Rigoni G., Raimondi A., Shintani N., Loureiro M., Vazquez J. (2016). Optic atrophy 1 is epistatic to the core MICOS component MIC60 in mitochondrial cristae shape control. Cell Rep..

[54] Bohnert M., Zerbes R.M., Davies K.M., Mühleip A.W., Rampelt H., Horvath S.E., Boenke T., Kram A., Perschil I., Veenhuis M. (2015). Central role of Mic10 in the mitochondrial contact site and cristae organizing system. Cell Metab..

[55] Ueda H.R., Hayashi S., Chen W., Sano M., Machida M., Shigeyoshi Y., Iino M., Hashimoto S. (2005). System-level identification of transcriptional circuits underlying mammalian circadian clocks. Nat. Genet..

[56] Ripperger J.A., Shearman L.P., Reppert S.M., Schibler U. (2000). CLOCK, an essential pacemaker component, controls expression of the circadian transcription factor DBP. Genes Dev..

[57] Zwighaft Z., Aviram R., Shalev M., Rousso-Noori L., Kraut-Cohen J., Golik M., Brandis A., Reinke H., Aharoni A., Kahana C., Asher G. (2015). Circadian clock control by polyamine levels through a mechanism that declines with age. Cell Metab..

[58] Magi B., Ettorre A., Liberatori S., Bini L., Andreassi M., Frosali S., Neri P., Pallini V., Di Stefano A. (2004). Selectivity of protein carbonylation in the apoptotic response to oxidative stress associated with photodynamic therapy: a cell biochemical and proteomic investigation. Cell Death Differ..

[59] Gong W., Zhou Y., Gong W., Qin X. (2020). Coniferyl ferulate exerts antidepressant effect via inhibiting the activation of NMDAR-CaMKII-MAPKs and mitochondrial apoptotic pathways. J. Ethnopharmacol..

[60] Jezek P., Hlavatá L. (2005). Mitochondria in homeostasis of reactive oxygen species in cell, tissues, and organism. Int. J. Biochem. Cell Biol..

[61] Kapałczyńska M., Kolenda T., Przybyła W., Zajączkowska M., Teresiak A., Filas V., Ibbs M., Bliźniak R., Łuczewski Ł., Lamperska K. (2018). 2D and 3D cell cultures - a comparison of different types of cancer cell cultures. Arch. Med. Sci..

[62] Giarmarco M.M., Brock D.C., Robbings B.M., Cleghorn W.M., Tsantilas K.A., Kuch K.C., Ge W., Rutter K.M., Parker E.D., Hurley J.B., Brockerhoff S.E. (2020). Daily mitochondrial dynamics in cone photoreceptors. Proc. Natl. Acad. Sci. USA.

[63] Ball J.M., Chen S., Li W. (2022). Mitochondria in cone photoreceptors act as microlenses to enhance photon delivery and confer directional sensitivity to light. Sci. Adv..

[64] Haverkamp S., Grünert U., Wässle H. (2000). The cone pedicle, a complex synapse in the retina. Neuron.

[65] Robinson J.T., Thorvaldsdóttir H., Winckler W., Guttman M., Lander E.S., Getz G., Mesirov J.P. (2011). Integrative genomics viewer. Nat. Biotechnol..

[66] Robinson J.T., Thorvaldsdóttir H., Wenger A.M., Zehir A., Mesirov J.P. (2017). Variant review with the integrative genomics viewer. Cancer Res..

[67] Thorvaldsdóttir H., Robinson J.T., Mesirov J.P. (2013). Integrative genomics viewer (IGV): high-performance genomics data visualization and exploration. Brief. Bioinform..

