# Supplementary material for: Bmal1 deletion alters mitochondrial microstructure and function in mouse cone photoreceptors
Source: iScience. 2025 Aug 22;28(9):113425. doi: 10.1016/j.isci.2025.113425 (PMC12455009; doi:10.1016/j.isci.2025.113425)

iScience, Volume *28*

Supplemental information

Bmal1 deletion alters mitochondrial microstructure and function in mouse cone photoreceptors

Nicolas Diaz, Sondip Biswas, Wei Zhong, Khaleel Bashir, Ting Chung Suen, Jason DeBruyne, Paul Michael Iuvone, Gianluca Tosini, Hao Duong, Sharon Francis, and Kenkichi Baba

Supplemental information

Figure S1. *in Silico* search of *Mic60* promoter binding assay.

Data mining on protein binding for the 10kb upstream of *Mic60* (*IMMT*) transcription site was done by ChIP-Atlas (Kyoto University in collaboration with DBCLS; https://chip-atlas.org/). The results showed two BMAL1, CLOCK binding sites close to the *Mic60* transcription start site (Peak Browser with IGV genome browser platform, q<0.05). The numbers under the binding sites indicate the bp lengths from *Mic60* transcription start site.

Supplemental Figure 1

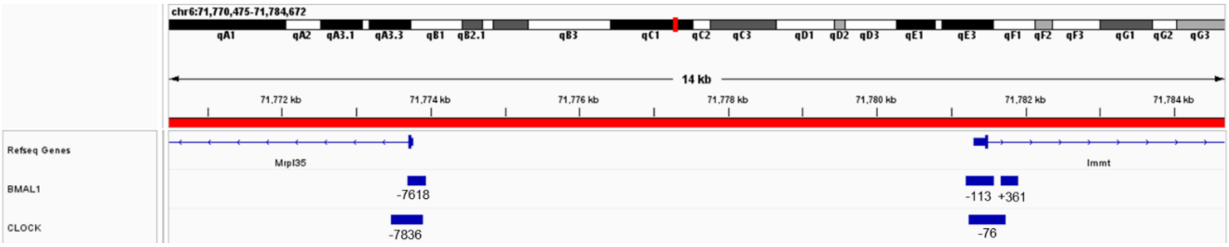

Supplement: Document S1. Figure S1 [file mmc1.pdf]
